# Functional connectivity of subsystems of the default-mode network in patients with early psychotic symptoms

**DOI:** 10.1016/j.ynirp.2025.100248

**Published:** 2025-03-11

**Authors:** Nicky Lute, Imke Lemmers-Jansen, Lydia Krabbendam, Mariët van Buuren

**Affiliations:** a Department of Clinical, Neuro and Developmental Psychology, Faculty of Behavioral and Movement Sciences. Vrije Universiteit Amsterdam, Van der Boechorststraat 7, 1081, BT Amsterdam, the Netherlands; b Institute for Brain and Behavior Amsterdam, Vrije Universiteit Amsterdam, Van der Boechorststraat 7, 1081, BT Amsterdam, the Netherlands

**Keywords:** Psychosis, Functional connectivity, Default mode network, Psychotic symptoms, Resting-state

## Abstract

Resting-state connectivity of the default-mode network (DMN) is aberrant in patients with chronic psychotic disorders as well in individuals with early stage psychosis. Studies of the DMN in healthy volunteers revealed that the DMN comprises several subnetworks. However, it is not yet clear if connectivity between and within these DMN subnetworks is aberrant in patients with early psychotic symptoms nor whether these connectivity patterns are related to symptomatology. This initial investigation examined functional connectivity between and within the DMN subnetworks in patients with early psychotic symptoms and in healthy volunteers, and probed how these connectivity patterns were related to the severity of clinical symptomatology. Functional connectivity was measured during resting-state in 30 patients with early psychotic symptoms and in 39 controls using functional MRI. We did not observe differences in connectivity within and between the subnetworks of the DMN between the control group and the early psychosis group. However, lower functional connectivity between the medial prefrontal and posterior medial subnetworks and between medial prefrontal and anterior temporal subnetworks of the DMN did predict the severity of the negative symptoms. The findings of this initial investigation provide insight into the associations between functional connectivity of DMN subnetworks and symptomatology in patients with early psychotic symptoms.

## 1 Introduction

Psychotic disorders are characterized by positive symptoms (e.g., delusions, hallucinations, disorganized speech), negative symptoms (e.g., affective flattening, lack of motivation, decreased social functioning) and cognitive impairments (Corcoran et al., 2011; Correll and Schooler, 2020; Fett et al., 2012; McCutcheon et al., 2020; Tandon et al., 2013). Our understanding of psychotic disorders and corresponding neural abnormalities has advanced throughout the years and previous neuroimaging research in patients with psychotic disorders as well as individuals at high-risk for developing a psychotic episode has shown functional and structural abnormalities in various brain regions (Barch and Ceaser, 2012; Fusar-Poli et al., 2011; Haijma et al., 2013; MacDonald et al., 2009; Owen et al., 2016; Woodward, 2016). Moreover, intrinsic functional connectivity of the brain appears to be affected in patients with chronic and early stage psychotic disorders (Kambeitz et al., 2016; Li et al., 2015, 2019; O'Neill et al., 2019; Satterthwaite and Baker, 2015), including individuals at high risk for developing a psychotic episode (Davies et al., 2024; Du et al., 2018; Lord et al., 2012).

A key resting state network that shows abnormal intrinsic connectivity in psychotic disorders is the default-mode network (DMN). The DMN is a large-scale network first described as a network of regions showing decreased activation during goal-directed tasks compared to rest (Raichle et al., 2001; Shulman et al., 1997) and its core regions include the ventral and dorsal medial prefrontal cortices, temporal parietal junction, posterior cingulate cortex, and temporal poles (Schurz et al., 2014, 2020). The DMN is associated with internally focused processing, including self-referential processing and autobiographical memory processes, as well as social cognitive processes (Buckner et al., 2008; Fox et al., 2005; Greicius et al., 2003; Gusnard and Raichle, 2001). It is suggested to play a central role in integrating internally self-related processing and external, social, information, important for establishing shared understanding (Yeshurun et al., 2021). Aberrant connectivity within the DMN may contribute to pathophysiology and symptomatology in patients with psychotic disorders, in particular related to difficulties in introspective processing, including an affected sense of self, and an altered link with the external world (Chan et al., 2021; Damiani et al., 2020; Northoff and Duncan, 2016; van Buuren et al., 2012). While a large body of literature has reported abnormal intrinsic connectivity of the DMN in patients, research has shown mixed findings, with both hypoconnectivity (meta-analyses (Dong et al., 2018; Li et al., 2019; O'Neill et al., 2019) and hyperconnectivity of the DMN (Damiani et al., 2020; Hu et al., 2017). These mixed findings in connectivity might be partly related to symptomatology as well as the stage of psychosis of the patients (early stage vs chronic) and associated effects of long-term illness and long-term medication use on connectivity. Research targeting the early stages of psychosis can provide more insight into the dysconnectivity of the DMN in psychosis and the association with symptomatology without suffering from these effects of long-term illness (Fusar-Poli et al., 2011).

Studies of the DMN in healthy volunteers revealed that the DMN is not a homogeneous network, but instead comprises several subnetworks related to different cognitive processes (Andrews-Hanna et al., 2010; Barnett et al., 2021; Buckner and DiNicola, 2019; DiNicola et al., 2020; Van Buuren et al., 2019). Various, but overlapping, fractionations of the DMN have been reported, including an anterior and posterior subdivision in healthy volunteers and patients with Alzheimer's disease (Damoiseaux et al., 2008, 2012), as well as a division into a core subnetwork related to the medial prefrontal cortex and posterior cingulate cortex, and a dorsal medial and a medial temporal lobe subsystem in healthy volunteers (Andrews-Hanna et al., 2010). In a recent study, the DMN was partitioned into three subnetworks; the posterior medial (PM), the anterior temporal (AT), and the medial prefrontal (MPF) subnetworks (Barnett et al., 2021). The PM subnetwork consists of the posterior cingulate cortex, retrosplenial cortex, lateral parietal and dorsal lateral prefrontal cortex and is thought to mediate information transfer in and out the DMN, and to represent episodic memory and the self (Barnett et al., 2021). The AT subnetwork comprises the temporopolar, lateral orbitofrontal and dorsal medial prefrontal cortices and is also believed to mediate information transfer, but is more associated with interpretations of theory of mind and social interactions (Barnett et al., 2021). Last, the MPF subnetwork includes the ventral medial prefrontal cortex and entorhinal cortex and is suggested to integrate information within the DMN and to represent emotional valence and perceived value of the event (Barnett et al., 2021; DiNicola et al., 2020). Investigating connectivity patterns within and between these DMN subnetworks may provide more insight in the pathophysiology of psychotic disorders. A small number of studies in patients with schizophrenia have examined connectivity within and between subnetworks of the DMN. Skudlarski and colleagues (Skudlarski et al., 2010) reported hyperconnectivity in chronic patients with schizophrenia within a DMN subnetwork encompassing the anterior posterior cortical midline structures, but not within a subnetwork comprising lateral regions, while another study in patients with schizophrenia (Wang et al., 2015) revealed hyperconnectivity between a subnetwork centered around the anterior regions of the DMN and a subnetwork of lateral regions of the DMN (at an uncorrected threshold). These studies provide initial support for aberrant connectivity of DMN subnetworks in patients with schizophrenia, however, it is not yet clear if connectivity between and within these DMN subnetworks is also affected in patients with early psychotic symptoms.

Connectivity of DMN subnetworks may be related to the severity of clinical symptoms. Studies targeting connectivity of the DMN in patients with early stage psychotic disorder without focusing on subnetworks showed that functional connectivity abnormalities between the DMN and regions outside the network were related to the severity of cognitive dysfunction (Wotruba et al., 2014) and positive symptoms (Hare et al., 2019; Lee et al., 2018; Manoliu et al., 2014; Yuan et al., 2022). Additionally, a meta-analysis reported hypoconnectivity of various regions of the DMN to be associated with the severity of negative symptoms in early stage psychosis (O'Neill et al., 2019). Together, these studies showed abnormal connectivity of the DMN depending on symptomatology in early stage psychosis. Additionally, Wang and colleagues (Wang et al., 2015) showed that connectivity between a subnetwork of lateral regions of the DMN and a network outside of the DMN (the right frontoparietal network) was positivity related to the severity of negative symptoms in patients with schizophrenia. The next step is to probe whether connectivity within and between DMN subnetworks specifically is related to symptomatology in patients with early psychotic symptoms.

In this initial investigation, we investigated functional connectivity between and within the DMN subnetworks in patients with early psychotic symptoms, including both patients who were at clinical high-risk for experiencing a psychosis and patients who experienced a first-episode psychosis (average time between diagnosis and study inclusion = 5.6 ± 4.9 months), and in healthy volunteers. Furthermore, we examined whether and how these connectivity patterns are related to clinical symptom severity. Functional connectivity was measured during resting-state in 30 patients with early psychotic symptoms and in 39 controls using fMRI. Clinical symptom severity was measured through an interview focusing on positive and negative symptoms experienced in the two weeks prior to the MRI session. We hypothesized that patients with early psychotic symptoms would display connectivity abnormalities within and between the subnetworks of the DMN compared to controls. However, as research targeting connectivity of the DMN subnetworks in early psychotic disorder is limited, we do not have expectations regarding the exact connectivity patterns. Furthermore, we expected that the presence of network abnormalities would be associated with the severity of clinical symptoms.

## 2 Materials and methods

### 2.1 Participants

The participants were selected from a larger study, of which results regarding neural correlates of trust and social decision-making have been previously published ((Lemmers-Jansen et al., 2020, 2019b, 2019a; Wisman-van der Teen et al., 2022). Of these participants, resting-state data were collected in 40 patients with early psychotic symptoms (mean age = 21.4 years, standard deviation [SD] = 2.7) and 47 healthy controls (mean age = 21.2 years, SD = 3.1). The early psychotic symptoms group consisted of 24 patients who experienced a first-episode psychosis (FEP) and 16 patients who were at clinical high-risk (CHR) for experiencing a psychosis. Of the included participants, 18 participants were excluded from data analyses (5 FEP, 5 CHR, 8 healthy controls); 1 participant (FEP) was excluded because of technical failure of the scanner, 15 participants (8 HC, 4 CHR and 3 FEP) were excluded because of motion (absolute motion above 3 mm or frame-wise displacement of >0.5 mm in more than 17% of the scans (see *2.4.2.2 Preprocessing*), 1 participant (CHR) was excluded because of low signal-to-noise ratio within the regions of the DMN, and 1 participant (FEP) was excluded because their functional connectivity values were statistical outliers (>3 SD). For the final group analyses, 39 healthy controls, 11 CHR and 19 FEP (n = 69) were included. The FEP patients were included within 18 months of the diagnosis (mean 5.6 ± 4.9 months) and the CHR were included within one year after assessment of psychotic experiences (mean 4.6 ± 3.7 months). For the analyses predicting psychotic symptoms, two more participants (CHR) were excluded because of missing symptom data, thus 28 participants (9 CHR and 19 FEP) remained as these analyses were conducted in the early psychotic symptoms group only. In all analyses, we combined the CHR and FEP patients because we were interested in intrinsic connectivity in patients with early psychotic symptoms. Moreover, by combining these patients, we increased the power of our analyses (see for similar approach (Lemmers-Jansen et al., 2020; Wisman-van der Teen et al., 2022)). The demographics of participants of the group comparison analysis (*n* = 69) and of the participants of the symptom analysis (*n* = 28) are shown in Table 1.

**Table 1:** Demographics. The psychosis group for symptom analyses (n = 28) is used to study the connection between functional connectivity and symptoms. The psychosis group for group analyses (n = 30) is used to compare functional connectivity with healthy controls. SD = standard deviation, WAIS = Wechsler Adult Intelligence Scale.

	Psychosis group (Symptom Analyses)	Psychosis group (Group Analyses)	Healthy controls
Gender (*n* male, %)	16 (57.10%)	16 (53.30%)	20 (51.30%)
Age (Mean/*SD*)	20.79 *(2.35)*	21.05 *(2.50)*	20.9 *(3.17)*
WAIS (Mean/*SD*)	38.64 *(12.81)*	38.07 *(12.56)*	43.28 *(11.27)*
Medicated (%)	13 (46.40%)	15 (48.40%)	–

The sample and recruitment procedure were also previously described in (Lemmers-Jansen et al., 2019a, 2019b, 2020). Patients who experienced a first-episode psychosis were diagnosed according to the DSMIV criteria, and included within 18 months of the diagnosis. Patients at clinical high-risk for psychosis were help-seeking individuals who underwent a semi-structured interview to assess psychotic experiences in the previous year (Comprehensive Assessment of At-Risk Mental States – CAARMS (Yung et al., 2005). Additionally, patients at clinical high-risk for psychosis displayed problems as measured on the Social and Occupational Functional Assessment Scale (Goldman et al., 1992, Morosini et al., 2000). Patients at clinical high-risk for psychosis were included within one year after CAARMS assessment. Healthy controls were randomly recruited at schools for secondary vocational education and matched based on the age and gender of the patient population. Exclusion criteria were an IQ < 80 and any contraindications for scanning. For the healthy control group, additional exclusion criteria were a (family) history of psychopathology, which was assessed with self-report, and by a systematic interview with questions regarding past and present mental help seeking, symptoms of depression and psychosis, and medication use. For the patient group, additional exclusion criteria were a primary diagnosis of mood disorders and comorbidity with autism spectrum disorder (ASD). The research was approved by the Ethics Committee of the VU Medical Center Amsterdam and carried out in accordance with the latest version of the Declaration of Helsinki. All participants provided written informed consent before participating in the study.

### 2.2 Materials

#### 2.2.1 Positive and negative syndrome scale (PANSS)

The PANSS is a 30-item semi-structured interview used for rating symptoms as experienced in the two weeks prior to testing. The PANSS distinguishes three types of symptoms: positive, negative, and general symptoms (Kay et al., 1987). Items are scaled on a 7-point Likert scale, where a rating of three or higher indicates a clinical value. Twenty-eight patients completed the PANSS.

#### 2.2.2 Wechsler adult intelligence scale (WAIS) vocabulary

As a proxy to measure intelligence, the vocabulary subtest of the WAIS-III was included (Wechsler, 1997). Verbal comprehension is measured by asking participants the definition or description of 33 words (e.g., winter, catastrophe, and reckless). Answers were either fully correct (2 points), partially correct (1 point) or wrong (0 points). After six consecutive wrong answers, the test was discontinued.

### 2.3 Procedure

When participants arrived at the research center, they were asked to sign three forms; general informed consent, one form that allowed the researchers to obtain additional patient data from their caregiving institution, and a pre-scanning questionnaire pertaining to the safety procedure for scanning and medication use. After signing the forms, participants completed questionnaires and the patients were assessed with the PANSS. Next, the participants were scanned for approximately an hour. In the scanner, all participants first performed two tasks which are described elsewhere (the Trust Game, see (Lemmers-Jansen et al., 2020, 2019a; Wisman-van der Teen et al., 2022)) and the SoMi task, see (Lemmers-Jansen et al., 2019b). Additionally, a structural scan was made during which participants were allowed to watch a short movie. Last, participants were scanned during a resting-state period of 6 min. During the resting-state, the stimulus screen was black and the participants were instructed to remain awake and lie still. After finishing the scan, participants were asked if they stayed awake, all participants confirmed that they remained awake.

### 2.4 Data analysis

#### 2.4.1 Demographic data

Demographic data were analyzed using RStudio (RStudio Team, 2019) with χ^2^ and t-tests. We analyzed group differences in age, gender and WAIS-scores between the psychosis group and the control group. Furthermore, between the patients at clinical high-risk for psychosis and patients who experienced a first-episode psychosis, we assessed differences in psychotic symptoms, as measured on the subscales of the PANSS, as well as medication use.

#### 2.4.2 Imaging data

##### 2.4.2.1 Data acquisition

MRI data were obtained at the Spinoza Center - Roeterseiland Amsterdam, using a 3.0 T Philips Achieva whole body scanner (Philips Healthcare, Best, Netherlands) equipped with a 32 channel head coil. A T2∗ EPI sequence (TR = 2, TE = 27.63, FA = 76.1°, FOV 240 mm, voxel size 3 × 3 × 3, 37 slices, 0.3 mm gap) was used to acquire data during a resting-state period of 6 min, resulting in 185 images. A T1-weighed anatomical scan was acquired for anatomical reference (TR = 8.2, TE = 3.8, FA = 8°, FOV 240 mm∗188 mm, voxel size 1 × 1 × 1, 220 slices).

##### 2.4.2.2 Preprocessing

The imaging data were preprocessed and analyzed using Statistical Parametric Mapping 12 (http://www.fil.ion.ucl.ac.uk/spm) and Matlab scripts. First, functional images were realigned to the reference image, followed by co-registration of the structural image to the mean functional image obtained after realignment. Next, unified segmentation was applied by segmentation of the co-registered structural image using tissue probability maps of SPM12, and subsequent normalization of the functional and structural images into Montreal Neurological Institute (MNI) space using the parameters estimated during segmentation. Last, smoothing was applied to the normalized functional images using a 3D Gaussian filter (6-mm full width at half maximum). Two healthy controls and two participants of the early psychosis group (one CHR and one FEP) were excluded because of motion, absolute displacement of more than one voxel (3 mm) (see *2.1. Participants*).

Next, to examine small scan-to-scan movement of the head that may affect connectivity, framewise displacement (FD (Power et al., 2012),) was calculated. A total of 11 participants (six healthy controls, five of psychosis group of which three CHR and two FEP) were excluded because of frame-wise displacement of >0.5 mm in more than 17% of the scan (see *2.1. Participants*). Of the remaining participants, the psychosis group (n = 30; FD = 0.23 mm) and healthy controls (n = 39; FD = 0.22 mm) did not differ in motion (*p* = .391). Motion scrubbing regressors were created to remove scans displaying FD-values of more than 0.5 mm, resulting in one regressor per outlier-scan. Next, these regressors, together with the six realignment parameters, average white matter and average cerebrospinal fluid signal were modeled in a voxel-wise multiple linear nuisance regression analysis to correct the normalized and smoothed functional imaging data for the effects of motion and non-regional specific signal changes. Additionally, a discrete cosine transform basis set was added to the regression to remove low-frequency fluctuations not of interest (>0.01 Hz). Residual functional imaging data after regression was used to calculate connectivity between and within the DMN subnetworks.

##### 2.4.2.3 Functional network parcellation

To derive a functional network parcellation, a method similar as described by van Buuren and colleagues was used (van Buuren et al., 2021). As a first step, the DMN was derived. Based on an existing brain parcellation (Power et al., 2011), 264 spherical nodes with a 5 mm radius were created. Regions with an average temporal signal-to-noise-ratio (tSNR) below 50 or an individual minimum below 30 were excluded. Furthermore, regions which the parcellation of Power termed as ‘uncertain’ were excluded, resulting in 205 nodes. Next, connectivity was calculated between all 205 nodes for each subject and averaged over participants. A modified Louvain clustering algorithm (1000 iterations) was applied to the resulting group averaged connectivity matrix to derive the networks (Brain Connectivity Toolbox (Power et al., 2011; Rubinov and Sporns, 2011)). Nodes assigned to a network in less than 40% of the iterations were removed from further analyses. For the detection of the DMN, a gamma of 1.25 was used resulting in 48 nodes assigned to the DMN. The normalized mutual information (NMI) between the obtained assignment vector and the original network assignment vector (Power et al., 2011) was NMI = 0.66.

**Fig. 1:**
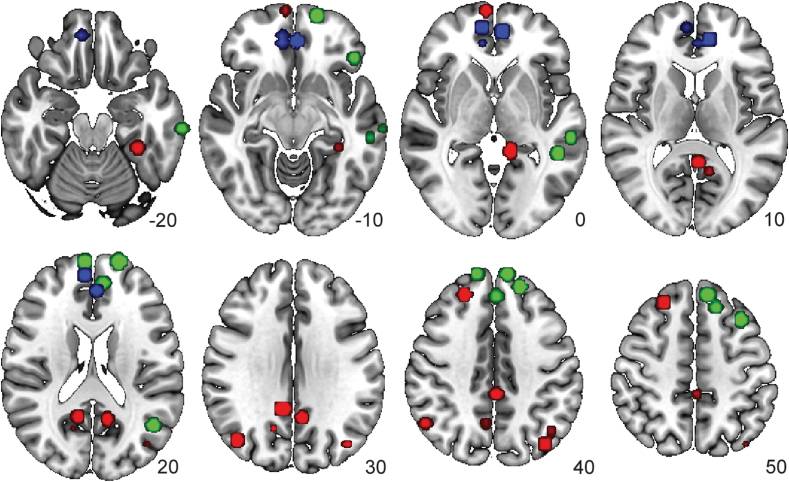
Nodes of the subnetworks of the DMN: posterior medial (PM; red), anterior temporal (AT; green), medial prefrontal (MPF; blue) represented on a template of standard brain (MNI152) with corresponding z-coordinates.

As a next step, the DMN subnetworks were derived (see (Barnett et al., 2021)). For each participant, the 48 nodes assigned to the DMN in the whole-brain network parcellation were selected and connectivity between these nodes was calculated and averaged across participants. A modified Louvain clustering algorithm with a gamma of 1.00 was applied to this matrix, resulting in the detection of three DMN subnetworks; PM, AT and MPF subnetwork (see Fig. 1).

##### 2.4.2.4 Functional connectivity analyses

Individual correlation coefficients within and between the three subnetworks were calculated and transformed to Fisher's z-scores, and compared between the healthy controls and the psychosis group, using two-sample independent t-tests. These analyses were repeated to compare the healthy controls to the psychosis group with symptomatology data (n = 28).

Next, to investigate whether connectivity between and within the networks could predict the severity of the psychotic symptoms, individual network connectivity values of the psychosis group (n = 28) were submitted to bootstrapping enhanced elastic net penalized least squares regression analyses (following (Abram et al., 2016; Bunea et al., 2011; van Buuren et al., 2021); R-code provided by (Abram et al., 2016) with the subscores of the PANSS as a dependent variable, and framewise displacement as co-predictor. Framewise displacement (FD) was included because it correlated significantly with positive symptoms of the PANSS (*r* = 0.39, *p* = .043). In these analyses, α was set to 0.5 to approach an elastic net. Note that the α does not represent the significance level, but determines whether the penalized regression approaches an elastic net, a lasso or ridge regression (Bunea et al., 2011). Predictors were selected when included in more than 70% of the bootstrap samples (5000), variable inclusion probability, VIP (Bunea et al., 2011; van Buuren et al., 2021). To estimate the resulting model and beta coefficients, selected predictors, as well as medication use as co-predictor (dummy coded), were submitted to an ordinary least square (OLS) multiple regression model, because penalized regression analysis causes a bias in estimating beta coefficients (Abram et al., 2016; Bunea et al., 2011).

##### 2.4.2.5 Exploratory functional connectivity analyses

To explore differences between CHR and FEP patients, and between these separate patient groups and healthy controls, exploratory two-sample independent t-tests were conducted contrasting connectivity within and between the DMN subnetworks between the CHR and FEP patients, and between each of these two patient groups and the healthy controls. Additionally, to explore potential differences between CHR and FEP patients in predicting the severity of symptoms, group (CHR/FEP) was added as a dummy coded co-predictor to the resulting ordinary least square (OLS) multiple regression model.

## 3 Results

### 3.1 Participant characteristics

Participants characteristics are shown in Table 1, Table 2. The early psychotic symptoms group (*n* = 30) and healthy controls (*n* = 39) did not differ significantly in gender (*p* = .866), age (*p* = .832), or WAIS scores (*p* = .074). The patients at clinical high-risk for psychosis (CHR) and the patients who experienced first-episode psychosis (FEP) did differ significantly in WAIS scores (*t*(26) = 2.62, *p* = .014) and age (*t*(26) = 4.36, *p* < .001), with higher WAIS scores and older age in the CHR group (see Table 2). The CHR and FEP did not significantly differ in gender (*p* = .080) or medication use (*p* = .885), or on positive symptoms (*p* = .088) or negative symptoms (*p* = .411) as measured with the PANSS (see Table 2). However, CHR did report significantly more general psychopathology symptoms (*t*(26) = 2.167, *p* = .040).

**Table 2:** Demographics and symptom severity in clinical high-risk patients and patients who experienced a first-episode psychosis. Symptoms were measured using the Positive and Negative Syndrome Scale (PANSS). CHR = clinical high-risk for psychosis, FEP = first-episode psychosis, n = sample size, WAIS = Wechsler Adult Intelligence Scale, SD = standard deviation.

	CHR (*n* = 9) mean (*SD)*	FEP (*n* = 19) mean (*SD)*
Gender (*n* male, %)	3 (33.33%)	13 (68.42%)
Age (Mean/*SD*)	22.98 *(2.14)*	19.77 *(1.65)*
WAIS (Mean/*SD*)	47.00 *(10.87)*	34.68 *(11.92)*
Medicated (%)	4 (44.44%)	9 (47.37%)
Positive symptoms	13.11 *(2.09)*	10.84 *(3.53)*
Negative symptoms	14.44 *(3.64)*	16.32 *(6.19)*
General psychopathology	33.00 *(6.40)*	27.47 *(6.26)*

### 3.2 Functional connectivity of the DMN subnetworks

#### 3.2.1 Network identification

The community detection algorithm resulted in three DMN subnetworks. The posterior medial (PM) network consisted of 18 nodes, the anterior temporal (AT) network consisted of 22 nodes, the medial prefrontal (MPF) network consisted of 8 nodes (see Fig. 1).

#### 3.2.2 Group differences in functional connectivity

Average functional connectivity values (Fisher's z-scores) within and between the subnetworks of the DMN are shown in Table 3. No significant differences were observed between the early psychosis and control group in connectivity within or between any of the DMN subnetworks. Similar findings were obtained when comparing the healthy controls to the early psychosis group with symptomatology data (n = 28). Moreover, exploratory two-sample t-tests contrasting connectivity within and between the DMN subnetworks between CHR, FEP, and between each of these patient groups and the healthy controls were conducted. No significant group differences were observed in any of the comparisons (see Supplementary Table 1).

**Table 3:** ***Functional connectivity values within and between the DMN subnetworks*.***Connectivity values are Fisher z-scores. Posterior medial (PM), anterior temporal (AT), medial prefrontal (MPF). n = sample size, t = t-value, p = p-value.*

Subnetworks DMN	Psychosis group (*n* = 30)	Healthy controls (*n* = 39)	Psychosis vs. Control
PM	0.45 *(0*.*10)*	0.47 *(0.09)*	*t*(67) = 0.65, *p* = .517
AT	0.39 *(0.08)*	0.40 *(0.09)*	*t*(67) = 0.71, *p* = .482
MPF	0.59 *(0.14)*	0.62 *(0.14)*	*t*(67) = 0.97, *p* = .337
MPF x PM	0.35 *(0*.*10)*	0.36 *(0.09)*	*t*(67) = 0.68, *p* = .497
PM x AT	0.27 *(0.09)*	0.28 *(0.07)*	*t*(67) = 0.60, *p* = .552
MPF x AT	0.33 *(0.08)*	0.34 *(0.11)*	*t*(67) = 0.70, *p* = .488

**Fig. 2:**
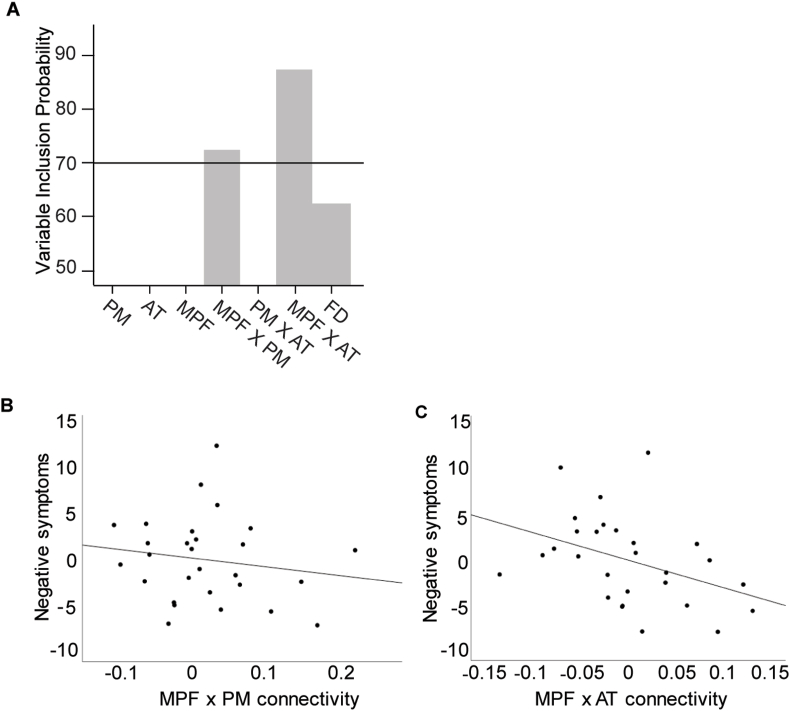
Connectivity predicting the severity of negative symptoms. A) Variable inclusion probability of the elastic net penalized regression, with the 70% threshold line. B) Lower connectivity between the MPF and PM and C) lower connectivity between the MPF and AT predicted more severe negative symptoms and together explained 29% of the variance in negative symptoms, R^2^ = 0.29.

Next, bootstrapping enhanced penalized regression analyses were performed to test the relation between functional connectivity and positive, negative, and general symptoms as measured with the PANSS. When applying a VIP threshold of 70%, two predictors were selected to explain individual variability in negative symptoms; connectivity between the MPF and PM, and connectivity between the MPF and AT (see Fig. 2A). In this resulting model, lower connectivity between the MPF and PM (β = −0.16) together with lower connectivity between the MPF and AT (β = −0.43), predicted more severe negative symptoms (see Fig. 2 B/C), and explained 29% of the variance in negative symptoms (R^2^ = 0.29, adjusted R^2^ = 0.23). To test the effect of the use of antipsychotic medication on symptom severity, medication use was added as a dummy variable to the model. Medication use did not contribute to explaining individual variability in symptom severity (R^2^ change = 0.018, *p* = .442, β = −0.15). Additionally, to explore potential differences between CHR and FEP patients in predicting the severity of negative symptoms, group (CHR/FEP) was added as a dummy coded co-predictor to the model. Group did not contribute to the model explaining negative symptom severity (R^2^ change = 0.009, *p* = .591, β = −0.10) (see Supplementary Materials for the resulting OLS regression model).

No predictors reached the VIP threshold when predicting severity of positive or general symptoms.

## 4 Discussion

In this initial investigation, we probed functional connectivity between and within the DMN subnetworks in patients with early psychotic symptoms and in healthy volunteers. Furthermore, we examined whether and how these connectivity patterns were related to clinical symptom severity. In contrast to our hypothesis, functional connectivity within and between the subnetworks of the DMN did not differ between the control group and the psychosis group. However, lower functional connectivity between the medial prefrontal (MPF) and posterior medial (PM) subnetworks and between MPF and anterior temporal (AT) subnetworks of the DMN did predict the severity of the negative symptoms in the early psychotic symptoms group. Connectivity within and between any of the DMN subnetworks did not explain the severity of positive or general symptoms.

Functional connectivity within and between subnetworks of the DMN did not differ between the control group and the early psychotic symptoms group. This is in contrast to our expectation and to two prior studies in patients with schizophrenia showing abnormal connectivity within and between DMN subnetworks (Skudlarski et al., 2010; Wang et al., 2015). In one of these studies, connectivity patterns between the subnetworks of the DMN were used to differentiate patients from healthy controls (Wang et al., 2015). Also, in that study, hyperconnectivity was reported (at an uncorrected threshold) between a DMN subnetwork comprising lateral parietal regions and a DMN subnetwork consisting of the medial prefrontal cortex and posterior midline and lateral regions in the patients with schizophrenia (Wang et al., 2015). In the other study, hyperconnectivity was reported within a DMN anterior posterior midline subnetwork, but not in a second DMN subnetwork in chronic patients with schizophrenia (Skudlarski et al., 2010). The lack of a group difference in the current study might be explained by the variability between individuals. Our early psychotic symptoms group consisted of both patients who experienced first-episode psychosis as well as patients at clinical high-risk. This latter group consisted of patients who reported psychotic symptoms, but had not experienced a full-blown psychosis, unlike the former group who did experience a first-episode psychosis (Lemmers-Jansen et al., 2019a). A recent study in chronic patients with schizophrenia showed differential connectivity patterns within the DMN depending on the clinical outcome (Lee et al., 2019). In that study, hypoconnectivity within the DMN was revealed in patients with a moderate and poor clinical outcome compared to healthy controls, while no differences in connectivity were observed when comparing patients with a good clinical outcome to the control group (Lee et al., 2019). Moreover, a recent longitudinal study in patients with early stage psychosis, who experienced a first psychotic episode with an onset within four years prior to the start of the study, showed no differences between the patients group and a group of healthy controls in change in connectivity of the DMN (Chan et al., 2021). However, they did observe differences in the change in DMN connectivity over time between patients with early stage psychosis depending on their outcome trajectory, with an increase in DMN connectivity in patients with worsening of symptoms and a decrease in connectivity over time in patients who showed improvement of symptoms (Chan et al., 2021). In the current study, exploratory analyses did not reveal any significant differences in connectivity patterns between patients who experienced a first-episode psychosis and the patients at clinical high-risk, however, this might be due to the small sample size (9 CHR and 19 FEP). For future research, more focus on functional connectivity within and between the DMN subnetworks in patients with early psychotic symptoms is recommended to shed light on potential differences in connectivity related to clinical outcome. Moreover, a longitudinal design could provide more insight into the development of these functional abnormalities when patients at clinical high-risk experience a first-episode psychosis.

While no differences in connectivity was observed between the early psychotic symptoms group and healthy controls, we did find that lower functional connectivity between the MPF and PM subnetworks and between MPF and AT subnetworks of the DMN predicted the severity of the negative symptoms. This is in line with a meta-analysis reporting hypoconnectivity of various regions of the DMN in association with severity of negative symptoms in early stage psychosis (O'Neill et al., 2019), however, severity of negative symptoms has also been related to hyperconnectivity in early stage psychosis (Chan et al., 2021). The current study adds to existing literature by showing that hypoconnectivity between specific subnetworks of the DMN is associated with the severity of symptoms, instead of hypoconnectivity of the DMN as a whole, or between specific regions. These subnetworks are believed to be implicated in different processes. That is, the MPF subnetwork is believed to be involved in integrating information within the DMN and representing emotional valence of an event, while the AT subnetwork is thought to mediate information transfer to and from the DMN as well as representing interpretations of theory of mind (Barnett et al., 2021). The PM is also suggested to play a role in information transfer into and from the DMN in addition to episodic memory processes (Barnett et al., 2021). We speculate that the observed lower connectivity between the MPF and the AT might result in less integration of the representation of the emotional valence of an event by the MPF and the interpretations of theory of mind by the AT. Lower connectivity between the MPF and the PM on the other hand may result in less integration of the representation of emotional valence by the MPF and episodic memory processes mediated by the PM. Together, this decreased exchange between the subnetworks and associated processes might contribute to negative symptoms such as blunted affect, emotional withdrawal, passive-apathetic social withdrawal. However, this interpretation is speculative and future research in large samples is required to further elucidate the association between connectivity between the MPF and AT and between the MPF and PM and negative symptoms in patients with early psychotic symptoms. More insight into the differentiation between the subnetworks of the DMN that show aberrant connectivity in relation to early psychotic symptoms may contribute to the understanding of the development of psychosis. However, further research is needed to determine whether and how such insight can be applied in clinical practice.

No association was found between connectivity within or between the DMN subnetworks and positive or general symptoms. While there are not many studies relating cognitive, or general symptoms to aberrant connectivity in patients with early psychotic symptoms (but see (Lee et al., 2018; Wotruba et al., 2014)), various studies did show abnormal connectivity associated with the severity of positive symptoms (Hare et al., 2019; Lee et al., 2018; Manoliu et al., 2014; Yuan et al., 2022). A key difference between these previous studies and the current study is that we focused on connectivity within and between DMN subnetworks, while in the previous studies, positive symptom severity was predominantly associated with abnormal connectivity between the DMN and salience network or central executive network (Hare et al., 2019; Lee et al., 2018; Manoliu et al., 2014). Possibly, positive symptoms are related to dysfunctional connectivity and interplay between networks, while negative symptoms are related to connectivity abnormalities within the DMN. This is in line with a study of Yuan and colleagues (Yuan et al., 2022), showing that positive symptoms were related to more cross-network connectivity abnormalities, while negative symptom severity was related to more focused network connectivity differences. However, given the small sample size of the current study, further research is required to replicate our lack of findings with regards to the severity of positive and general symptoms and functional connectivity within and between DMN subnetworks.

A possible limitation of the present study is the medication use of the patient group. Previous research has shown that antipsychotic medication can increase connectivity in certain parts of the brain, including the frontal and parietal lobes (Bolding et al., 2012; Zong et al., 2019), although other sudies revealed no differences between patients receiving medication and non-medicated patients (Wang et al., 2015). In our analyses, we controlled for the use of medication, but only whether a participant used medication or not. Unfortunately, we did not have information regarding the dose of medication, or how long medication was used. Relatedly, while connectivity between subnetworks of the DMN explained part of the severity of negative symptoms, no association was found between connectivity and positive or general symptoms. Possibly, antipsychotic medication is overall more effective for positive symptoms, and side-effects might even exacerbate (the severity of) negative symptoms (Lally and MacCabe, 2015; Tandon, 2011). Last, variability between the clinical high-risk and first-episode psychosis patients may have resulted in the absence of connectivity differences when compared to healthy controls. While both clinical high-risk and first-episode patients experienced psychotic symptoms, different mechanisms might underlie the behavioral outcomes and symptomatology (Van Os and Reininghaus, 2016). Future studies are required to probe potential differences in connectivity patterns between patients with early psychotic symptoms. Furthermore, patients who are at a clinical high-risk of experiencing a psychosis are already in care for other psychopathology, including anxiety and depression, which may have contributed to the heterogeneity in our participant sample.

In sum, in this initial investigation, we did not observe overall differences in connectivity within and between the DMN subnetworks between patients with early psychotic symptoms and healthy volunteers. However, we did find that that lower connectivity between the MPF and PM and between the MPF and PM subnetworks of the DMN was associated with more severe negative symptoms. While future research is required to further examine this association, this study provides insights into the associations between functional connectivity of DMN subnetworks and symptomatology in patients with early psychotic symptoms. Also, these findings contribute to understanding the mixed findings of abnormal connectivity within the DMN in patients with early stages of psychosis.

## CRediT authorship contribution statement

**Nicky Lute:** Writing – original draft, Formal analysis, Conceptualization. **Imke Lemmers-Jansen:** Writing – review & editing, Resources, Project administration, Investigation. **Lydia Krabbendam:** Writing – review & editing, Funding acquisition. **Mariët van Buuren:** Writing – review & editing, Writing – original draft, Supervision, Methodology, Formal analysis, Conceptualization.

## Funding

This work was supported by funding of the 10.13039/501100008358Hersenstichting Nederland [KS 2011(1)-75], a VIDI and VICI grant from the 10.13039/501100003246Dutch Research Council (10.13039/501100003246NWO) (452-07-007 and 453-11-005), and an ERC Consolidator grant (648082 SCANS) to LK.

## Declaration of competing interest

None.

## Data Availability

The authors do not have permission to share data.
