# Supplementary material for: Functional connectivity of subsystems of the default-mode network in patients with early psychotic symptoms
Source: Neuroimage Rep. 2025 Mar 11;5(1):100248. doi: 10.1016/j.ynirp.2025.100248 (PMC12172822; doi:10.1016/j.ynirp.2025.100248)
Supplement: Supplementary file 1 — Multimedia component 1 [file mmc1.pdf]

Supplementary Results

	**CHR (*n* = 11)**	**FEP (*n* = 19)**	**CHR vs. HC**	**FEP vs. HC**	**CHR vs. FEP**
PM	0.49 *(0.09)*	0.44 *(0.10)*	*p* = .614	*p* = .224	*p* = .193
AT	0.40 *(0.06)*	0.38 *(0.09)*	*p* = .824	*p* = .443	*p* = .674
MPF	0.58 *(0.15)*	0.60 *(0.14)*	*p* = .387	*p* = .485	*p* = .783
MPF x PM	0.36 *(0.10)*	0.34 *(0.10)*	*p* = .866	*p* = .414	*p* = .667
PM x AT	0.30 *(0.08)*	0.25 *(0.09)*	*p* = .548	*p* = .223	*p* = .196
MPF x AT	0.34 *(0.09)*	0.32 *(0.08)*	*p* = .925	*p* = .389	*p* = .501

Supplementary Table 1. *Functional connectivity values within and between the DMN subnetworks in the early psychosis group.* *Values are reported separately for clinical high-risk patients and patients who experienced a first-episode psychosis. Connectivity values are Fisher z- scores. Exploratory comparisons between groups were performed using independent t-tests. Posterior medial (PM), anterior temporal (AT), medial prefrontal (MPF). n = sample size, p = p-value.*

Resulting OLS regression model

To estimate the resulting model and beta coefficients, selected predictors, as well as medication use as co-predictor (dummy coded), were submitted to an ordinary least square (OLS) multiple regression model. The resulting model was:

- Severity of negative symptoms= Intercept + Connectivity MPF x PM* β_1 + Connectivity MPF x AT * β_2. R^2 = .29.

Next, to test for effects of medication use, a dummy coded predictor was added:

- Severity of negative symptoms= Intercept + Connectivity MPF x PM* β_1 + Connectivity MPF x AT * β_2 + medication use * β_3. ΔR^2 = .018.

Last, to explore for effects of group (CHR or FEP), a dummy coded predictor was added:

- Severity of negative symptoms= Intercept + Connectivity MPF x PM* β_1 + Connectivity MPF x AT * β_2 + medication use * β_3 + patient group * β_4. ΔR^2 = .009.
